# Disrupted Lipid Metabolism, Cytokine Signaling, and Dormancy: Hallmarks of Doxorubicin-Resistant Triple-Negative Breast Cancer Models

**DOI:** 10.3390/cancers16244273

**Published:** 2024-12-23

**Authors:** Radhakrishnan Vishnubalaji, Nehad M. Alajez

**Affiliations:** 1 Translational Oncology Research Center (TORC), Qatar Biomedical Research Institute (QBRI), Hamad Bin Khalifa University (HBKU), Qatar Foundation (QF), Doha P.O. Box 34110, Qatar; vbradhakrishnan@hbku.edu.qa; 2 College of Health & Life Sciences, Hamad Bin Khalifa University (HBKU), Qatar Foundation (QF), Doha P.O. Box 34110, Qatar

**Keywords:** triple-negative breast cancer (TNBC), doxorubicin, chemoresistance, cellular dormancy, therapeutic strategies

## Abstract

Background: Chemoresistance in triple-negative breast cancer (TNBC) presents a significant clinical hurdle, limiting the efficacy of treatments like doxorubicin. This study aimed to explore the molecular changes associated with doxorubicin resistance and identify potential therapeutic targets to overcome this resistance, thereby improving treatment outcomes for TNBC patients. Methods: Doxorubicin-resistant (DoxR) TNBC models (MDA-MB-231 and BT-549) were generated by exposing cells to increasing concentrations of doxorubicin. RNA sequencing (RNA-Seq) was performed using the Illumina platform, followed by bioinformatics analysis with CLC Genomics Workbench and iDEP. Functional assays assessed proliferation, sphere formation, migration, and cell cycle changes. Protein expression and phosphorylation were confirmed via Western blotting. Pathway and network analyses were conducted using Ingenuity Pathway Analysis (IPA) and STRING, while survival analysis was performed using Kaplan–Meier Plotter database. Results: DoxR cells exhibited reduced proliferation, sphere formation, and migration, but showed enhanced tolerance to doxorubicin. Increased CHK2 and p53 phosphorylation indicated cellular dormancy as a resistance mechanism. RNA-Seq analysis revealed upregulation of cytokine signaling and stress-response pathways, while cholesterol and lipid biosynthesis were suppressed. Activation of the IL1β cytokine network was prominent in DoxR cells, and CRISPR-Cas9 screens data identified dependencies on genes involved in rRNA biogenesis and metabolism. A 27-gene signature associated with doxorubicin resistance was linked to worse clinical outcomes in a large breast cancer cohort (HR = 1.76, FDR *p* < 2.0 × 10^−13^). Conclusions: This study uncovers potential therapeutic strategies for overcoming TNBC resistance, including dormancy reversal and targeting onco-ribosomal pathways and cytokine signaling networks, to improve the efficacy of doxorubicin-based treatments.

## Simple Summary

This research focuses on understanding why some triple-negative breast cancers (TNBC) become resistant to chemotherapy, particularly doxorubicin, which is a major obstacle in treatment. The study, by developing models of doxorubicin-resistant TNBC cells, has uncovered that these cells enter a dormant-like state, slow their growth, and activate specific survival pathways. These resistant cells also show changes in metabolism and signaling, particularly involving cytokines, lipid metabolism, and ribosomal function. In identifying these mechanisms, the study suggests new treatment approaches that could target these survival strategies to make chemotherapy more effective and potentially improve patient outcomes.

## 1. Introduction

Triple-negative breast cancer (TNBC) exhibits the highest mortality rate among breast cancer subtypes due to its heterogeneity, aggressiveness, and poor differentiation compared to hormone receptor-positive (HR+) and HER2+ tumors, resulting in a lack of standardized treatment regimens [1]. According to Lehmann et al. differential gene expression and ontology profiling of TNBC tumors revealed six subtypes: basal-like (BL1 and BL2), mesenchymal (M), mesenchymal stem-like (MSL), immunomodulatory (IM), luminal androgen receptor (LAR), and unspecified unstable tumors (UNS) [2]. Other classification approaches to resolve TNBC heterogeneity have been proposed [3]. TNBC subtypes exhibit varying responses to neoadjuvant chemotherapy (NAC), with the BL1 subtype showing the highest pathologic complete response (pCR) to carboplatin-containing regimens, whereas LAR subtypes (excluding MSL) were found to be less sensitive [4].

Taxanes and anthracyclines are the main chemotherapeutic regimen for TNBC; however, less than 30% of TNBC patients achieve a complete response, and mortality and recurrence rates remain higher compared to non-TNBC subtypes [1]. Despite chemotherapy being the primary systemic treatment, resistance often develops, and the molecular and cellular properties of the relapse-prone subset of TNBC remain elusive. Employing single-cell transcriptome analysis, we previously demonstrated substantial tumor heterogeneity among TNBC cases and immune signatures correlating with the response to NAC [5]. This underscores the clinical challenge of chemoresistance in TNBC and the necessity of delineating resistance mechanisms and developing novel treatment approaches.

Doxorubicin, an anthracycline, is effective against both solid and non-solid tumors and is typically administered for node-positive breast cancer [6,7]. Doxorubicin is known to inhibit DNA synthesis, inhibit topoisomerase II, and induce free radical production and lipid peroxidation [8,9]. The addition of paclitaxel after cyclophosphamide and doxorubicin improves survival and reduces relapse rates in early breast cancer [10]. Meta-analyses show that anthracycline plus taxane regimens result in a 14% lower recurrence rate compared to taxane without anthracycline, with significant reductions in recurrence and mortality [11].

Several mechanisms have been identified that facilitate drug resistance, including mutations or changes in drug target expression, enhanced DNA damage repair, activation of alternative signaling pathways, and evasion of cell death [12]. Resistance can be inherent or acquired, with cancer dormancy facilitated by quiescence and tumor mass dormancy. Understanding quiescent cancer cell biology has led to therapeutic strategies such as awakening dormant cells for chemotherapy, maintaining dormancy, or eliminating dormant cells [13]. Resistant subsets of TNBC may enter a quiescent, drug-tolerant persister (DTP) state, enabling them to survive chemotherapy and later resume proliferation [14,15].

To further understand TNBC resistance mechanisms, we established a doxorubicin-resistant (DoxR) model to study dormancy pathways. Our study contributed to the mechanisms by which TNBC evades doxorubicin therapy and achieves dormancy and suggested potential therapeutic approaches to enhance treatment efficacy and overcome resistance.

## 2. Materials and Methods

### 2.1. Cell Line Authentication Through Short Tandem Repeat (STR) Analysis

To authenticate the MDA-MB-231 and BT-549 TNBC cell lines, the AmpFLSTR Identifiler PCR amplification kit from Thermo Fisher Scientific, Inc. (Waltham, MA, USA) was used for genomic DNA (gDNA) amplification. Following amplification, the PCR products underwent preparation for electrophoresis by adding Hi-Di Formamide and a size-standard mixture to each sample and allelic ladder. Electrophoresis was performed using the Genetic Analyzer 3500xl DX system (Applied Biosystems, Carlsbad, CA, USA). Allelic calls analysis was conducted using the Gene Mapper Release 6.1 software provided by Applied Biosystems. Reference STR profiles were obtained from the American Type Culture Collection (ATCC; https://www.atcc.org/, accessed on 10 October 2024).

### 2.2. Cell Culture and Generation of DoxR Cell Models

Human TNBC cell lines MDA-MB-231 and BT-549 were cultured in DMEM (Dulbecco’s modified Eagle’s medium) supplemented with 10% fetal bovine serum (FBS) and 1% penicillin/streptomycin purchased from Gibco, Waltham, MA, USA. The cells were maintained at 37 °C with 5% CO_2_ in a humidified incubator. We generated doxorubicin-resistant TNBC cell lines by subjecting the cells to repeated, gradually increasing concentrations of doxorubicin (1–15 nM) over a period of >12 months, as part of a continuous drug exposure regimen.

### 2.3. CFU Assay and Drug Toxicity Detection Using Fluorescence Microscopy

On day 7, cells were washed and stained with crystal violet (0.5% in 20% EtOH) at room temperature. After 10 min, plates were washed, and images were captured and compared with controls. CFU quantification was performed by measuring the absorbance of dissolved crystal violet in 5% SDS at 590 nm. The experiments were repeated at least twice, and data were presented as the mean ± SD from four replicates. Additionally, the AO/EtBr fluorescence staining method was used to assess dead and live cells. At the indicated time points, cells were stained with a dual fluorescent staining solution (100 μg/mL AO and 100 μg/mL EtBr) and imaged under a fluorescence microscope to identify viable and dead cells [16].

### 2.4. Organoid Dome and Spheroid Cultures

To create three-dimensional (3D) organoids, cell pellets (250,000 cells/mL) were combined with overnight-thawed Matrigel (Corning, Corning, NY, USA; 356231; Growth Factor Reduced (GFR) Basement Membrane Matrix). Multiple drops of the cell suspension were then plated in pre-warmed (37 °C) 60 mm Ultra-Low Attachment Culture Dishes (Corning; 3261). The dishes were placed upside down in a 37 °C, 5% CO_2_ cell culture incubator for 20 min to allow the droplets to solidify. After solidification, 4–5 mL of expansion medium was added, and the dishes were further incubated. Organoid formation was observed under a microscope after one week. Tumor spheroids were generated in 60 mm low cell binding dishes (Nunc; Thermo Fisher Scientific). DoxR and parental TNBC cells were trypsinized from monolayers and transferred to the dishes. Spheroid formation was initiated with 10,000 cells. On day 10, established spheroids were analyzed and stained with acridine orange and ethidium bromide.

### 2.5. Scratch Assay

To assess the migration of DoxR and parental TNBC cells, the cells were trypsinized and reseeded in 6 cm cell culture dishes. Once the cells reached confluence, the cell monolayers were scratched using a plastic micropipette tip (yellow tip; 20–200 μL). The cell monolayers were washed, and the medium was replaced with fresh culture medium. Images of the wounded region were taken immediately (0 h) and after 24 h using phase-contrast microscopy. Quantification of wound areas was conducted using ImageJ (https://www.tessonics.com/products/imagel/, accessed on 10 November 2024). 

### 2.6. Cell Cycle Analysis Using Flow Cytometry (FACS)

Cell cycle analyses were performed on parental and DoxR MDA-MB-231 and BT-549 TNBC cells in the absence or presence of the indicated doxorubicin concentrations. Cells were fixed with 70% ice-cold ethanol, stained with RNase A and propidium iodide (PI), and subjected to cell cycle analysis using BD LSR Fortessa X-20 flow cytometer at the FL3 channel.

### 2.7. Evaluation of Early and Late Apoptosis

Apoptosis was evaluated using the Annexin V-FITC/PI detection kit (ab14085; Abcam, Cambridge, UK) following the manufacturer’s protocol. In brief, 100,000 cells were washed with PBS, resuspended in binding buffer, and incubated with 5 µL of Annexin V and 5 µL of PI (50 µg/mL) at room temperature for 5 min in the dark. Apoptosis was then analyzed using the FL1 and FL2 channels on a Beckman Coulter CytoFLEX flow cytometer to quantify apoptotic cells.

### 2.8. Total RNA Library Preparation and RNA-Seq Analysis

Total RNA samples with a RIN > 8 were used for library preparation using the TruSeq Stranded Total RNA Library Prep Gold kit from Illumina. The library preparation involved rRNA depletion, fragmentation, first-strand cDNA synthesis with random hexamers and SuperScript II Reverse Transcriptase, and second cDNA strand synthesis with dUTP substitution. The resulting double-stranded cDNA was end-repaired, adenylated, and ligated with barcoded DNA adapters. After amplification, the library’s quality was assessed on an Agilent 2100 Bioanalyzer system and quantified using a Qubit 2.0 fluorometer. The libraries were pooled, clustered, and sequenced on an Illumina HiSeq 4000 platform at a minimum of 50 million paired-end reads (2 × 75 bp) per sample. Paired-end FASTQ files were aligned to the GRCh38 reference genome using the built-in module and default settings in CLC Genomics Workbench v23.0. Expression data (total counts) were then imported into iDEP.951, where they were first normalized (CPM, counts per million) and then transformed using EdgeR (log2(CPM + c)). DESeq2 was used to identify differentially expressed genes (DEGs) in DoxR compared to parental cells (2.0 FC and <0.05 FDR).

### 2.9. Gene Set Enrichment Analysis (GSEA) and Modeling of Gene Interaction

Differentially expressed genes from the RNA-seq analysis were imported into the IPA software version 127006219 (Qiagen Inc., Redwood City, CA, USA). Functional regulatory networks and canonical pathways were determined using URA, downstream effects analysis (DEA), MNs, and casual network analysis (CNA) prediction algorithms. IPA uses a precise database to model functional regulatory networks from a list of individual genes and determines a statistical score, the Z score, for each network based on the fit of the network to the set of focus genes. The biological functions assigned to each network are ranked according to the significance of that biological function to the network.

### 2.10. Protein–Protein Interaction (PPI) Network Analysis

Downregulated genes in DoxR TNBC models were subjected to protein–protein interaction (PPI) analysis using the STRING database v 11.5 to predict potential interactions among upregulated genes with a minimum interaction score of 0.4. GeneMANIA was used to assess enriched associations among DoxR 27-gene signatures essential for TNBC. 

### 2.11. Western Blotting

For immunoblotting experiments, DoxR and parental cells were lysed using RIPA buffer containing HALT protease and a phosphatase-inhibitor cocktail. Western blotting was performed using primary and secondary antibodies from the Cell Cycle/Checkpoint Antibody Sampler Kit (cat. no. 9917, Cell Signaling Technology, Boston, MA, USA). Membranes were visualized using SuperSignal™ West Femto ECl substrate and the ChemiDoc XRS+ Gel Imaging System. Band intensities were analyzed using Image Laboratory 5.0 software.

### 2.12. Survival Analysis

Kaplan–Meier survival analysis of potential therapeutic targets was conducted using the KMplot database. Mean expression values of selected genes were used to generate KM plots for all breast cancer patients and those with the TNBC subtype.

### 2.13. Identification of DoxR-Associated TNBC-Essential Genes

Genome-wide CRISPR-Cas9 functional screen data were retrieved from the Achilles project. An average gene effect score of ≤−0.5 was used as the cut-off for the 22 TNBC models.

### 2.14. Statistical Analysis

Statistical analyses were performed using Microsoft Excel and GraphPad Prism 9.0 software. IDEP.961 was used for differential expression analysis with a 2.0-fold change and FDR *p* < 0.1. For gene expression analysis, a two-tailed *t*-test was used, with *p* < 0.05 considered significant.

## 3. Results

### 3.1. Establishment and Characterization of DoxR TNBC Models

**Figure 1:**
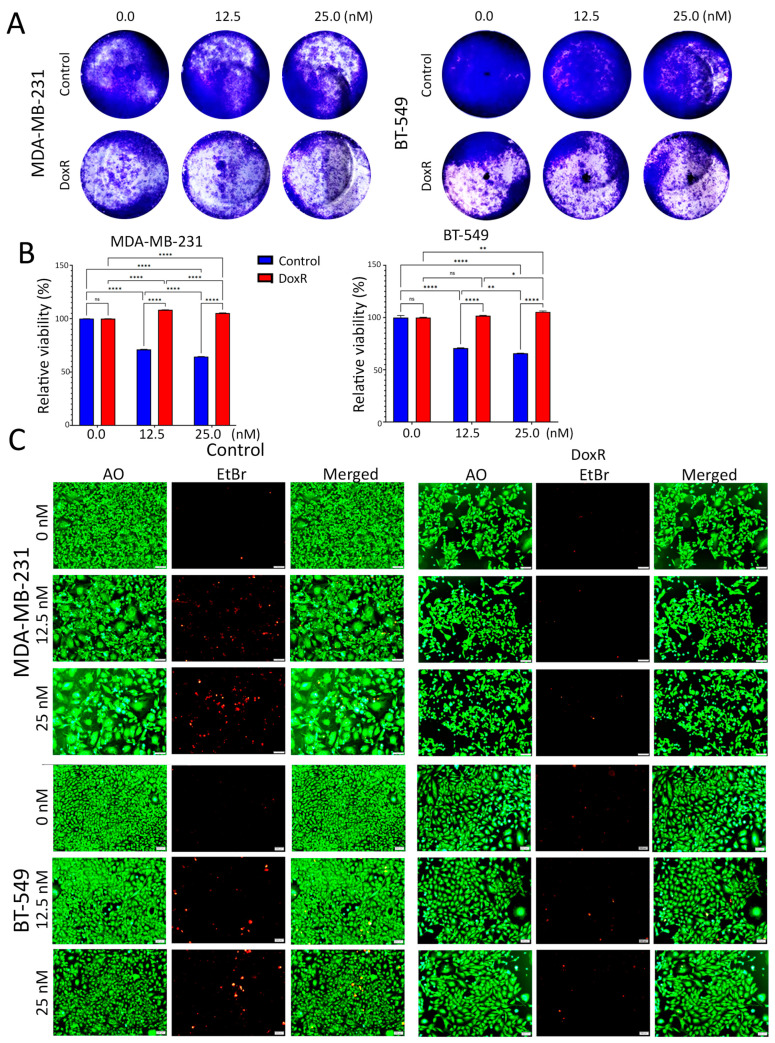
Proliferation and morphological changes in parental and DoxR TNBC cells following doxorubicin treatment. (**A**) Clonogenic survival assay (CFU) showing dose-dependent suppression of proliferation in parental TNBC cells (MDA-MB-231 and BT-549) treated with 12.5 nM and 25 nM doxorubicin, while DoxR cells maintained higher proliferation rates. (**B**) Quantitative analysis of CFU potential reveals reduced inhibition of cell proliferation in DoxR MDA-MB-231 and BT-549 cells compared to parental cells at both doxorubicin concentrations. Data are presented as mean  ±  S.E.M., n  =  6. n.s., not significant; * *p*  <  0.05; ** *p*  <  0.005; **** *p*  <  0.00005. Two-way ANOVA testing was employed. (**C**) Acridine orange/ethidium bromide (AO/EtBr) staining of parental and DoxR cells, demonstrating the presence of necrotic cells (red) and morphological abnormalities in response to doxorubicin in parental cells.

To gain insight into the complex mechanisms of doxorubicin resistance in TNBC, we generated DoxR models and characterized their cellular phenotype and molecular profiles. Parental and DoxR cells were treated with 12.5 nM and 25 nM concentrations of doxorubicin. Employing the CFU assay (day 7), the data presented in Figure 1A showed a dose-dependent suppression, with around 70% proliferation at 12.5 nM and 65% at 25 nM in parental cells, whereas DoxR cells exhibited sustained proliferation under the same conditions. Quantitative analysis of relative CFU potential revealed less inhibition of cell proliferation in DoxR MDA-MB-231 and BT-549 cells compared to parental cells under the indicated doxorubicin concentration (Figure 1B). Notably, DoxR cells were less proliferative compared to their parental counterpart. In parallel, DoxR and parental cells were stained with acridine orange/ethidium bromide (AO/EtBr) to evaluate their sensitivity to doxorubicin. Although DoxR cells were less proliferative compared to parental cells, DoxR cells exhibited greater tolerance, especially at higher concentrations of doxorubicin. Notably, there were remarkable differences in cellular morphology between DoxR and parental cells in response to doxorubicin treatment. Parental cells lost their normal features, exhibited an abnormal phenotype, and stained positive for EtBr, indicating necrotic cell death (Figure 1C).

### 3.2. Suppressed Functions Indicating Dormant State in DoxR TNBC Models

**Figure 2:**
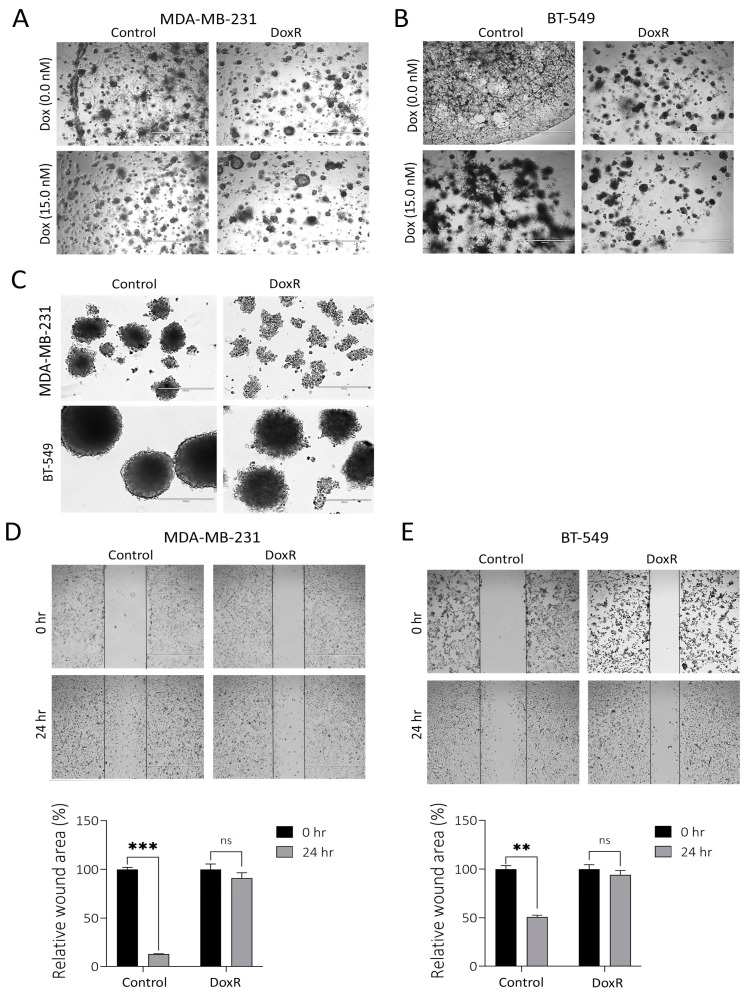
Suppressed migration and organotypic growth in DoxR TNBC models. Representative organotypic images for MDAMB-231 (**A**) or BT-549 (**B**) DoxR and control TNBC cells. (**C**) Spheroid formation assay showing reduced spheroid formation capacity in DoxR TNBC cells compared to parental cells. Migration assay illustrates a significant reduction in the migration of DoxR MDA-MB-231 (**D**) and BT-549 (**E**) cells compared to parental cells. Quantification of relative wound areas (%) are shown below. Data are presented as mean ± S.D., n = 2. n.s., not significant; ** *p*  <  0.005; *** *p*  <  0.0005.

We assessed the organotypic growth of parental and DoxR TNBC models under 3D culture conditions using extracellular matrix (ECM) cultures. A significant decline in the proliferation and migration of DoxR MDA-MB-231 and BT-549 cells was observed compared to parental cells under these conditions, respectively (Figure 2A,B). In the absence of doxorubicin, parental TNBC cells developed short cytoplasmic protrusions resembling invadopodia, forming a cobweb-like pattern. This characteristic was absent in DoxR cells, which failed to form the cobweb pattern even when exposed to doxorubicin (Figure 2A,B). Additionally, DoxR TNBC cells demonstrated reduced spheroid formation compared to parental cells (Figure 2C). The scratch assay further revealed diminished migration potential in DoxR cells compared to parental TNBC cells (Figure 2D,E). Overall, our data indicates that DoxR TNBC cells exhibit reduced organotypic growth, spheroid formation, and migration, suggesting altered cellular behavior.

### 3.3. Cell Cycle Regulation in DoxR TNBC Cells

**Figure 3:**
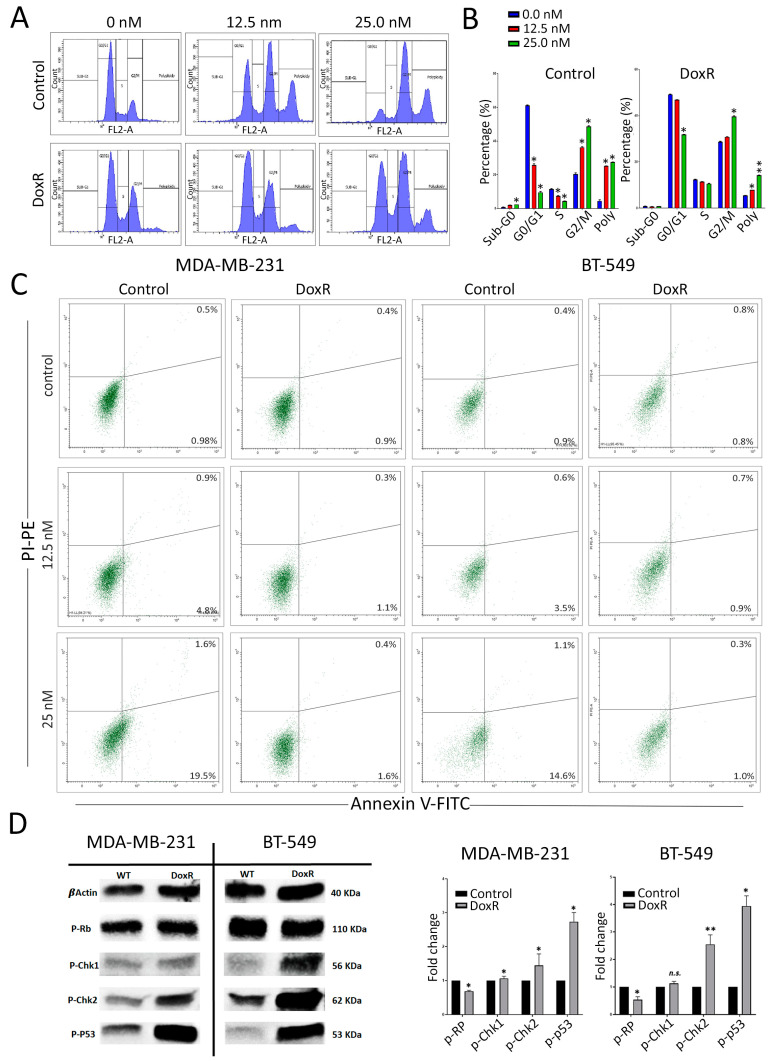
Alterations in cell cycle regulation in DoxR TNBC cells. (**A**) Flow cytometry analysis of cell cycle distribution in parental and DoxR cells post-doxorubicin treatment on day 3. (**B**) Quantification of cell cycle distribution form (**A**). Data are presented as mean ± S.D., n = 2. (**C**) Annexin V staining of control and DoxR TNBC cells at the indicated doxorubicin concentrations on day 3. (**D**) Representative Western blot analysis showing phosphorylated Retinoblastoma protein (p-Rb), phosphorylated ChK kinases, and p53 in DoxR TNBC cells. Quantifications of band intensity are shown on the right panel. Data are presented as mean ± S.D., n = 2. n.s., not significant; * *p* < 0.05; ** *p* < 0.005.

We subsequently utilized flow cytometry to examine the cell cycle distribution in parental and DoxR cells following doxorubicin treatment. Our findings revealed a significant inhibition of cellular proliferation in parental cells, as evidenced by a decrease in the cell population in the G0/G1 and S phases, along with an increase in arrest in the G2/M phase and polyploidy, particularly at higher doxorubicin concentrations (Figure 3A,B). However, this effect was less pronounced in DoxR TNBC cells. Annexin V staining revealed induction of apoptosis under higher doxorubicin concentrations in parental cells and, to a lesser extent, in DoxR cells (Figure 3C). To gain insights into the cell cycle progression and DNA integrity during the transitions from G1/S to G2/M in DoxR cells under cellular stress, we analyzed molecular circuit checkpoints. We observed a downregulation of phosphorylated Retinoblastoma (p-Rb) protein and an upregulation of phosphorylated ChK kinases and p53 in DoxR-TNBC cells (Figure 3D). These alterations in the cell cycle process suggest that DoxR cells enter a dormant state, contributing to their resistance to doxorubicin treatment.

### 3.4. Molecular Profiling of DoxR TNBC Models

**Figure 4:**
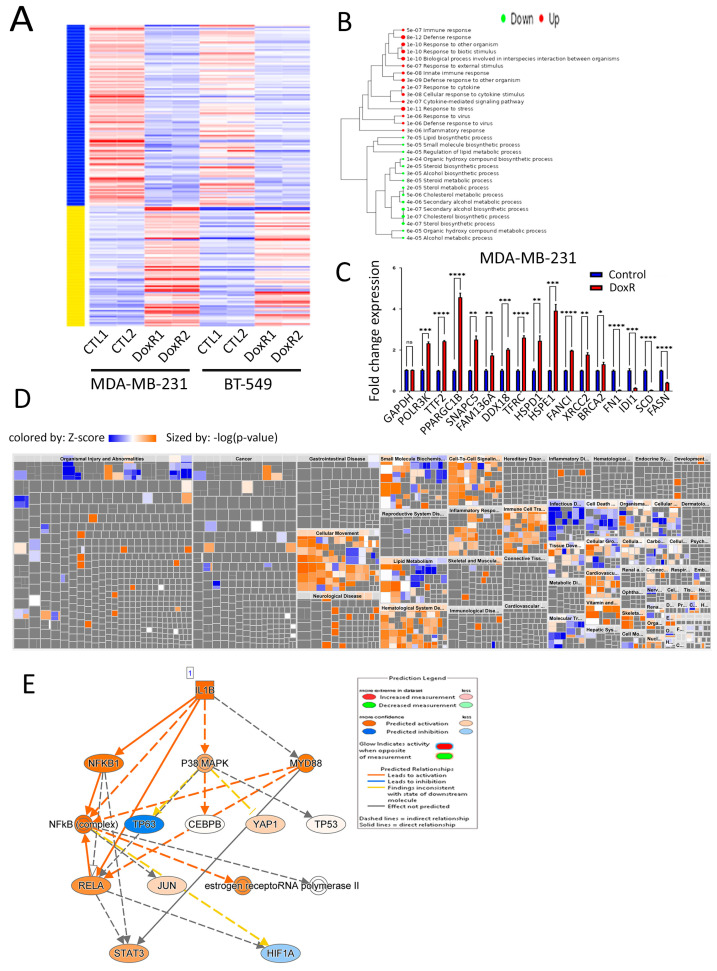
Molecular profiling of DoxR TNBC models. (**A**) Heatmap of differentially expressed genes (DEGs) showing 231 upregulated and 420 downregulated genes in DoxR cells compared to parental TNBC models. (**B**) Hierarchical clustering of enriched gene ontology (GO) biological processes, with upregulated genes associated with defense and immune responses and downregulated genes involved in cholesterol synthesis. (**C**) RT-qPCR validation of selected upregulated and downregulated genes in DoxR MDA-MB-231 cells. Data are presented as mean  ±  S.E.M., n = 9. * *p*  <  0.05, *** *p*  <  0.0005. (**D**) Disease and function heatmap depicting activated (orange) and suppressed (blue) categories in DoxR vs. control TNBC cells. (**E**) Upstream regulator analysis illustrating IL1β as a key driver of gene expression changes in DoxR TNBC models. n.s., not significant; ** *p* < 0.005; **** *p* < 0.00005.

Differential expression analysis identified 231 upregulated and 420 downregulated genes in DoxR compared to parental TNBC models (2.0-fold-change (FC) and <0.1 false discovery rate (FDR) adjusted *p*-value (Figure 4A, Table S1). Hierarchical clustering of enriched GO biological process terms revealed that upregulated genes are associated with defense, stress, and immune responses while downregulated genes were predominantly involved in cholesterol synthesis (Figure 4B). The expression of selected upregulated and downregulated genes was validated using RT-qPCR in MDA-MB-231 (Figure 4C) and BT-549 (Figure S1) DoxR TNBC cells. STRING Protein–Protein Interaction Networks functional enrichment analysis on downregulated genes revealed that cholesterol and sterol biosynthesis, as well as the negative regulation of lipid biosynthesis, were among the top suppressed biological processes in DoxR TNBC cells (Table S2). Illustrations of the cholesterol biosynthesis network among commonly downregulated genes in both DoxR models employing STRING analysis are shown in Figure S2. Ingenuity Pathway Analysis (IPA) on differentially expressed genes (DEGs) revealed strong enrichment in numerous canonical pathways. In agreement with GO analysis, we observed a notable downregulation of pathways related to lipid metabolism, cell death and survival, and cell growth and proliferation, alongside an upregulation of immune cell trafficking and inflammatory response (Figure 4D). Interestingly, upstream regulator analysis identified the activation of the IL1β molecule as a key driver of gene expression changes observed in DoxR TNBC models (Figure 4E). Correlation analysis with clinical outcomes based on the median expression of the top 100 downregulated genes indicated better recurrence-free survival (RFS) in breast cancer patients (Figure S3), thus corroborating our findings.

### 3.5. Dependency Map of DoxR TNBC Models Highlights a Role for Ribosomal RNA

**Figure 5:**
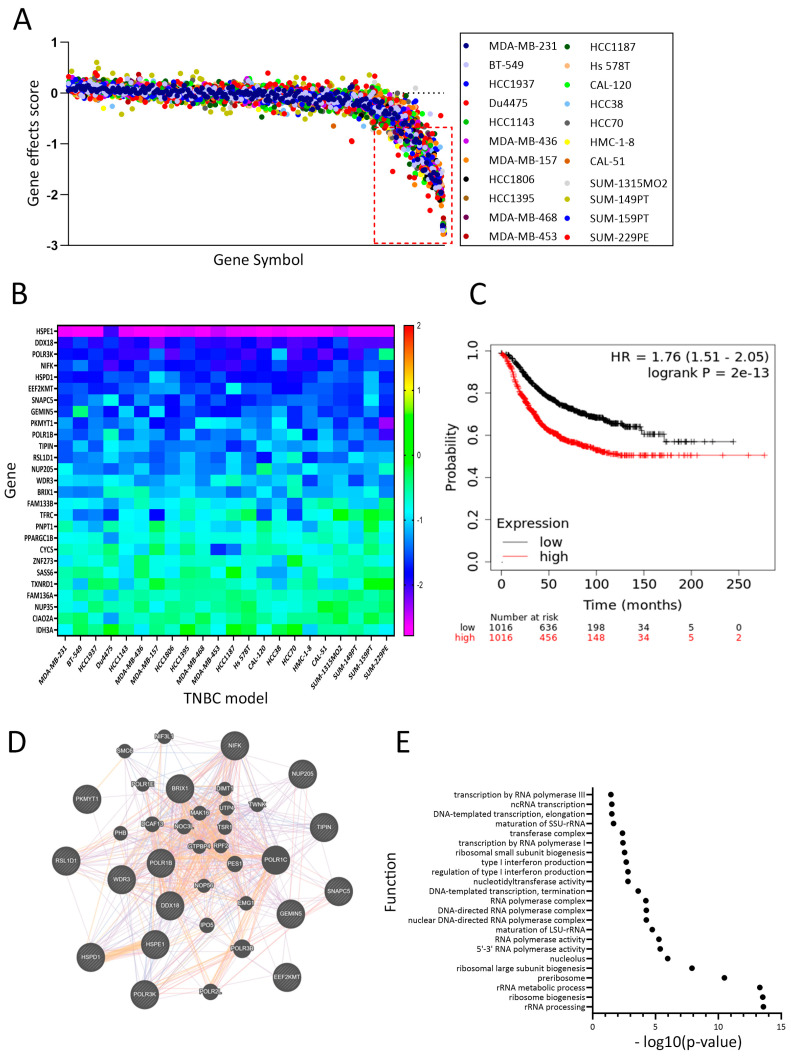
Dependency map of DoxR TNBC models highlighting the role of ribosomal RNA. (**A**) CRISPR-Cas9 functional screen data from DepMap identifying essentiality of 27 genes among 211 upregulated genes in DoxR TNBC models. (**B**) The effects of each identified gene in the panel of 22 TNBC models are depicted as a heatmap. The heatmap represents the dependency scores calculated by DepMap for the identified genes. (**C**) Kaplan–Meier plot showing that the identified 27-gene signature is associated with poor relapse-free survival (RFS) in breast cancer patients. (**D**) GeneMANIA analysis showing significant enrichment of the identified genes in functional networks associated with rRNA biogenesis and metabolism, underscoring their critical role in TNBC pathophysiology. (**E**) Functional enrichment among the DoxR-derived signature employment GeneMANIA.

Interrogation of upregulated genes in DoxR and the list of genes essential for TNBC survival employing CRISPR-cas9 functional screen data from DepMap [17] in a panel of 22 TNBC cell models using a cut-off of −0.5 (red line) revealed essentiality of 27 genes among the 231 commonly upregulated genes in DoxR TNBC models (Figure 5A). The effects of each identified gene in the panel of 22 TNBC models are depicted as a heatmap, with HSPE1 exhibiting the highest TNBC dependency (Figure 5B). The identified 27-gene signature was associated with poor relapse-free survival (RFS) in breast cancer patients (Figure 5C). GeneMANIA analysis of genes with a gene effect score of <−1.0 revealed significant enrichment in functional networks associated with rRNA biogenesis and metabolism (Figure 5D,E). These findings highlight the critical role of the identified gene signature in TNBC pathogenesis and underscore its potential as a therapeutic target, emphasizing the significance of rRNA biogenesis and metabolism pathways in driving poor clinical outcomes.

## 4. Discussion

TNBC poses significant clinical challenges due to its high rates of relapse, metastasis, and limited therapeutic options, resulting in a poorer prognosis compared to other breast cancer subtypes. While chemotherapy remains the mainstay of TNBC treatment, the development of resistance to anthracyclines and taxanes further complicates disease management. Efforts to address TNBC heterogeneity and resistance have led to numerous clinical trials investigating targeted therapies, including those targeting receptors such as EGFR, PARP inhibitors, androgen receptor (AR), estrogen receptor ER-α36, and immunotherapy agents like PD-L1 inhibitors and CAR-T therapy [18,19,20,21,22,23]. However, despite advancements in targeted treatments, cytotoxic chemotherapy remains the primary therapeutic approach for TNBC, with variable outcomes observed among patients with similar clinicopathologic characteristics. 

The complexity of TNBC resistance mechanisms, including both genetic and non-genetic factors, underscores the importance of understanding the mechanisms driving chemotherapeutic resistance, with potential utilization to stratify patients for appropriate treatment regimens and rational drug combinations. The molecular mechanisms driving resistance transformation and entry into a dormant state remain incompletely understood. Doxorubicin is an anthracycline-based chemotherapeutic agent commonly used to treat patients with TNBC. The mechanisms underlying doxorubicin’s pharmacodynamics in cancer cells involve its conversion to an unstable metabolite, semiquinone, by enzymes such as NAD(P)H quinone dehydrogenase 1 (NQO1), nitric oxide synthases (NOS3), and xanthine oxidase (XDH), followed by reconversion to doxorubicin, releasing reactive oxygen species (ROS). ROS then induce lipid peroxidation, membrane damage, and ultimately cell death through DNA damage and oxidative stress. Additionally, doxorubicin can induce cell death by directly entering the nucleus and disrupting the topoisomerase-II-mediated DNA repair mechanism involving genes such as TOP2A, MLH1, MSH2, ERCC2, and TP53 [24]. Despite its clinical value, resistance to doxorubicin poses limitations to its efficacy. Previous data have shown numerous resistance mechanisms involving various pathways, including activation of the MAPK/ERK pathway and the involvement of genes such as MMP1, VIM, CNN3, LDHB, NEFH, PLS3, AKAP12, TCEAL2, ABCB1 (MDR1), ABCC1 (MRP1), ABCC2, ABCC3, ABCG2, and RALBP1 [24,25]. Doxorubicin resistance has also been associated with amplification of TOP2A and ERBB2 (HER-2), which interfere with treatment response. Notably, TOP2A and HER-2 co-occur on chromosome 17 q12–q21, along with BRCA1 and P53, which play significant roles in breast cancer carcinogenesis [26,27,28,29].

In this study, we established DoxR TNBC models to investigate the signaling pathways associated with transient resistance and the dormancy-like state. Functional assays in 2D and 3D systems elucidated the impact of doxorubicin on tumorigenic properties, highlighting the importance of utilizing biologically relevant models to study chemoresistance. Our findings demonstrated significant inhibition of cell proliferation, migration, and sphere formation in DoxR cells, suggesting their entry into a dormant state in response to chemotherapeutic challenge. Our findings are concordant with Rehman et al. in their study delineating how colorectal cancer cells adopt a reversible drug-tolerant persister (DTP) state to evade chemotherapy and targeted treatments, contributing to therapy failure and tumor relapse. Using cellular barcoding and mathematical modeling, the authors have shown that DTPs are not confined to a small subset of cells but rather arise across the entire cancer cell population. Interestingly, these DTPs exhibit similarities to diapause, a reversible state of suspended embryonic development triggered by adverse environmental conditions [30]. Additionally, our analysis revealed dysregulation of key proteins involved in cell cycle checkpoints, indicating a shift towards dormancy in DoxR TNBC cells. Notably, DoxR cells exhibited significant inhibition of cell proliferation, migration, and sphere formation. Unlike parental TNBC cells, which showed notable arrest in the G2M phase upon exposure to doxorubicin, this response was less pronounced in DoxR TNBC models, suggesting that these cells enter a dormant state in response to chemotherapeutic challenges. Subsequent investigation into the effects of doxorubicin on key proteins associated with cell cycle checkpoints revealed reduced levels of p-Rb and increased levels of p-Chk1, p-Chk2, and p-P53 in DoxR cells. CDK1 (Cdc2) plays a crucial role in regulating the G2M shift, and its inhibition can prevent the phosphorylation of the tumor suppressor protein retinoblastoma (RB), thereby blocking the transcription of proliferative genes mediated by E2F [31,32]. Additionally, p53, a key player in the G1 checkpoint, activates G1 following DNA damage induced by doxorubicin during transient mitotic arrest [33]. Cells lacking functional p53 can bypass the p53 checkpoint and continue progressing through the cell cycle [34]. Active p53 stimulates the transcription of downstream target genes, including p21, which suppresses CDKs. Loss of p53 function frequently promotes carcinogenesis and is a prevalent trait in many cancers [35,36].

Our data revealed alteration in cholesterol and mevalonate-associated genes as the hallmark of DoxR TNBC, suggesting a metabolic dormancy state in TNBC cells. These findings align with previous research implicating the mevalonate pathway in cancer progression and drug resistance. Considering the pivotal role of cholesterol and mevalonate in cancer progression, particularly in promoting a high proliferation rate and metabolic shifting, as well as contributing to drug resistance via the stimulation of the estrogen-related receptor alpha (ERRα) pathway [37], it is imperative to explore their involvement in cancer drug resistance. The mevalonate pathway has garnered attention as a potential therapeutic target due to its role in synthesizing sterols like cholesterol and isoprenoids [38]. Central to this pathway is 3-hydroxy-3-methylglutaryl-CoA reductase (HMGCR), which catalyzes the conversion of 3-hydroxy-3-methylglutaryl-CoA into mevalonate and is considered the rate-limiting enzyme [39]. Elevated expression of HMGCR has been associated with a poorer prognosis and more aggressive tumors in various cancers, including breast cancer [40]. Our observation of downregulated cholesterol and mevalonate-associated genes such as HMCGS1, MVD, MVK, TM7SF2, LSS, FDFT1, DHCR24, IDI1, MSMO1, and INSIG1 in the DoxR TNBC model suggests a state of slow proliferation and lower metabolic dormancy in TNBC cells.

Metabolic alterations and active factors secreted by senescent cells, known as the senescence-associated secretory phenotype (SASP), play a critical role in chemotherapy resistance and cancer development [41]. Inflammatory cytokines within the SASP, such as IL1β, are believed to be essential contributors to chemoresistance, as we have reported in a current study. Recent research indicates that tumor cells can directly produce IL1β, rendering treatments ineffective [42]. IL1β has been implicated in docetaxel resistance by regulating the development of polyploid large cancer cells in non-small cell lung cancer [43]. In breast cancer, IL1β has a complex role in bone metastasis, suppressing growth in primary tumors while promoting the growth of osteolytic metastases. Combining doxorubicin and zoledronic acid with the IL-1 receptor antagonist Anakinra has shown promise in decreasing the growth of primary tumors and their metastases in syngeneic breast cancer models [44]. Our investigation of upstream regulators identified activation of the IL1β cytokine network in DoxR cells, along with enrichment of pathways linked to cell invasion, colony formation, and lipid metabolism. The potential role of downregulated lipid metabolism in contributing to chemotherapeutic resistance is an area that warrants deeper investigation. Alterations in lipid metabolism can influence cancer cell survival, membrane composition, energy homeostasis, and signaling pathways, all of which may impact the efficacy of chemotherapy. Understanding the mechanisms by which suppressed lipid metabolism promotes resistance could uncover novel therapeutic targets, improve drug sensitivity, and enhance treatment outcomes in resistant cancers.

The integration of CRISPR-Cas9 functional screens highlighted TNBC dependencies on metabolism-associated genomic networks and rRNA biogenesis, further emphasizing the multifaceted nature of TNBC resistance mechanisms. In DoxR TNBC models, RNA-Seq data revealed overexpression of RNA polymerase transcriptomes such as POLR1B, POLR3K, and POLR3G, as well as ribosomal protein RPS27L. Single-cell RNA sequencing showed high expression of RPL15 and RPL27A in metastatic TNBC, with silencing of RPL27A inhibiting migration and invasion [45]. RPS27L, primarily found in the cytoplasm under normal circumstances, undergoes dynamic translocation to the nucleus in response to DNA damage or ribosomal stress, where it stabilizes p53 by inhibiting MDM2-induced p53 ubiquitination. Conversely, silencing of RPS27L reduces p53 levels and inhibits its transcriptional activity. Concordantly, dormant cell cycle arrest is characterized by dysregulation of ribosomal biogenesis, which inhibits cell proliferation [46,47]. Aberrations in key tumor suppressor pathways, such as RB and p53, intensify nucleolar function and trigger nucleolar enlargement [48]. Moreover, p53-independent cell cycle arrest can occur in response to impaired ribosome biogenesis [49]. Perturbations in ribosome synthesis can lead to nucleolar stress and activation of the RP-Mdm2-p53 stress response pathway [50]. Continued synthesis of new rRNA is essential for ribosome biogenesis. Dysregulation of ribosome synthesis can lead to biogenesis stress, hyperactivation of oncogenic translation programs, and chemotherapy resistance. Emerging evidence suggests that cancer cells harbor oncogenic ribosomes, which may drive tumor growth and heterogeneity [47]. Additionally, modified rRNA has been identified as an oncogenic driver that can initiate tumor growth and contribute to heterogeneity [51]. While our data provide a thorough characterization and molecular profiling of the TNBC in vitro doxorubicin resistance model, further investigation using in vivo models is necessary to contextualize our findings and confirm their clinical relevance.

## 5. Conclusions

Our study sheds light on cellular dormancy, suppression of fatty acid metabolism, activation of cytokine signaling, and rRNA biosynthesis as hallmarks associated with DoxR TNBC cell models. Therapeutic strategies targeting reversal of the dormant-like state or suppression of onco-ribosomal networks and cytokine signaling pathways could enhance the efficacy of doxorubicin-based therapeutics and pave the way for overcoming TNBC resistance.

## Data Availability

All data are provided as supplementary data. Additional data can be provided upon request.

## Supplementary Materials

The following supporting information can be downloaded at: https://www.mdpi.com/article/10.3390/cancers16244273/s1, Figure S1: RT-qPCR validation of selected upregulated and downregulated genes in DoxR BT-549 cells; Figure S2: Illustration of cholesterol biosynthesis pathway in downregulated genes in doxorubicin-resistant (DoxR) TNBC, Figure S3: Correlation analysis of the top 100 downregulated genes in DoxR cells with poor recurrence-free survival (RFS) in breast cancer patients; Table S1: Differentially expressed genes in DoxR vs. control TNBC cells (2.0 FC, *p* < 0.05 FDR), Table S2: Enriched GO categories in downregulated genes in DoxR compared to control TNBC cells.
