# Supplementary material for: Disrupted Lipid Metabolism, Cytokine Signaling, and Dormancy: Hallmarks of Doxorubicin-Resistant Triple-Negative Breast Cancer Models
Source: Cancers (Basel). 2024 Dec 23;16(24):4273. doi: 10.3390/cancers16244273 (PMC11674486; doi:10.3390/cancers16244273)
Supplement: Supplementary file 1 [file cancers-16-04273-s001.zip › Figure S1.pdf]

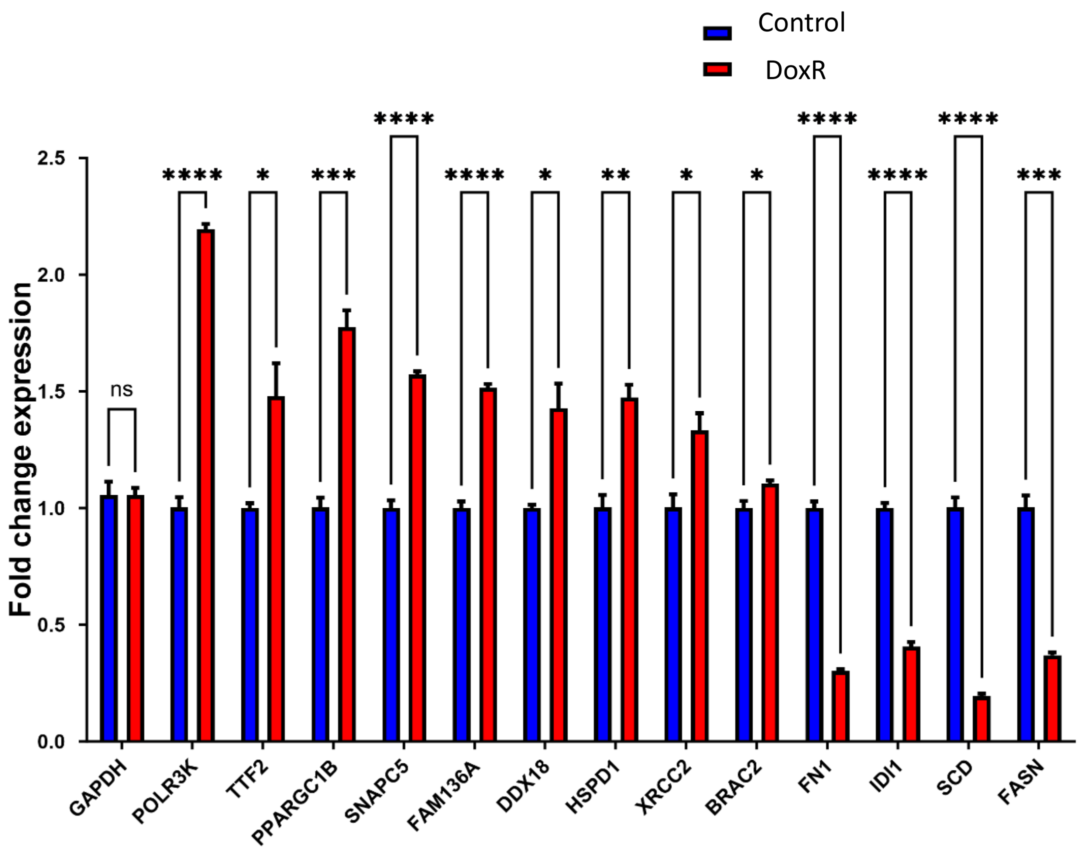

Figure S1. RT-qPCR validation of selected upregulated and downregulated genes in DoxR BT-549 cells. Data are presented as mean ± S.E.M., n=9. $^*p < 0.05$, $^{***}p < 0.0005$.
