# Supplementary material for: Disrupted Lipid Metabolism, Cytokine Signaling, and Dormancy: Hallmarks of Doxorubicin-Resistant Triple-Negative Breast Cancer Models
Source: Cancers (Basel). 2024 Dec 23;16(24):4273. doi: 10.3390/cancers16244273 (PMC11674486; doi:10.3390/cancers16244273)
Supplement: Supplementary file 1 [file cancers-16-04273-s001.zip › Figure S3.pdf]

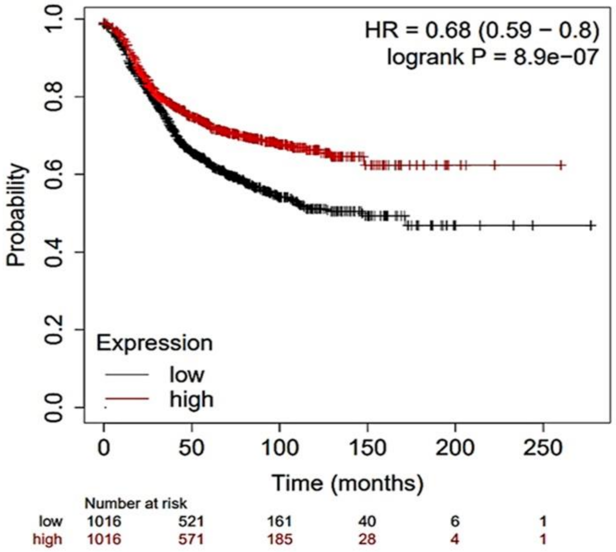

Figure S3. Correlation analysis of the top 100 downregulated genes in DoxR cells with poor recurrence-free survival (RFS) in breast cancer patients.
